# Comprehensive Analysis of the TLP Gene Family in Pine and Functional Implications in Response to Pine Wood Nematode Infection

**DOI:** 10.3390/biology15110878

**Published:** 2026-06-02

**Authors:** Yibo An, Ping Luo, Shengyin Xiao, Chao Pan, Huyi Zhou, Xuyang Wang, Yun Xiao, Minghui Guo

**Affiliations:** 1 College of Material Science and Engineering, Northeast Forestry University, 26 Hexing Road, Harbin 150040, China; aybnefu@163.com; 2 Chongqing Key Laboratory of Forest Ecological Restoration and Utilization in the Three Gorges Reservoir Area, Chongqing 400036, China; 3 Chongqing Key Laboratory of Tree Germplasm Innovation and Utilization, Chongqing 400715, China; 4 Chongqing Rare and Unique Fish National Nature Reserve Management Office, Chongqing 402260, China

**Keywords:** thaumatin-like proteins, *Bursaphelenchus xylophilus*, disease resistance, gene regulatory network

## Abstract

Pine wilt disease, caused by *Bursaphelenchus xylophilus*, poses a serious threat to global pine forest ecosystems and forestry production. Thaumatin-like proteins (TLPs), which belong to the PR-5 family, are known to participate in plant defense, but their roles in pine have not been well characterized. In this study, a comprehensive genome-wide analysis of the TLP gene family was conducted in *Pinus taeda*. A total of 116 TLP genes were identified and classified into four major clades based on phylogenetic analysis. Gene structure and conserved motif analyses revealed that members within the same clade generally exhibited similar exon–intron organization patterns and conserved motif compositions. Promoter analysis identified numerous cis-regulatory elements associated with stress responses and phytohormone signaling. Transcriptome data from different stages of pine wood nematode infection identified eight TLP genes that exhibited continuous differential expression, and their expression patterns were further confirmed by qRT-PCR. A multilayer regulatory network highlighted MYB and other transcription factors as key upstream regulators, and yeast one-hybrid assays confirmed MYB-mediated regulation. Together, these findings improve our understanding of the TLP gene family in *P. taeda* and offer valuable candidate genes and regulatory information for future studies on pine resistance to pine wilt disease.

## Simple Summary

Pine wilt disease is a serious forest disease that causes extensive damage to pine trees and leads to major ecological and economic losses worldwide. Thaumatin-like proteins (TLPs) are known to participate in plant defense, but their roles in pine have not been well characterized. In this study, we identified 116 TLP genes from the *Pinus taeda* genome and analyzed their structural characteristics, evolutionary relationships, and expression patterns during pine wood nematode infection. Eight TLP genes exhibited strong and continuous responses following infection, suggesting that they may play important roles in pine defense. Regulatory network analysis further indicated that MYB transcription factors may function as key upstream regulators involved in TLP-mediated defense responses. This study provides the first comprehensive analysis of the TLP gene family in pine and offers valuable candidate genes for future research on disease resistance. These findings may contribute to the development of resistant pine varieties and support sustainable management strategies for pine forests.

## 1. Introduction

Pine wilt disease (PWD) is a highly destructive forest disease caused by the *Bursaphelenchus xylophilus* [1,2]. Owing to its rapid transmission and high mortality rate, it is often referred to as “pine cancer” [3]. Although extensive studies have been conducted on the pathogenic mechanisms of PWD, the molecular basis underlying host resistance and defense responses remains incompletely understood. Current studies suggest that *B. xylophilus* infection disrupts host physiological processes through multiple pathways, including secretion of cell wall-degrading enzymes, induction of toxic metabolite accumulation, and promotion of xylem dysfunction [4]. These pathological processes are closely associated with plant immune responses, suggesting that defense-related proteins may play important roles during nematode infection.

Within the sophisticated and multilayered plant immune system, pathogenesis-related proteins (PRs) constitute an important chemical defense barrier against pathogen invasion [5]. Among these, thaumatin-like proteins (TLPs), which belong to the PR-5 family, have attracted considerable attention because of their broad involvement in plant resistance to fungi, bacteria, viruses, and various abiotic stresses [6]. The functional versatility of TLPs is closely associated with their highly conserved and stable molecular architecture [7]. A defining structural characteristic of TLPs is the presence of 16 conserved cysteine residues forming eight intramolecular disulfide bonds, which stabilize their three-dimensional structure [6]. This compact structure enables TLPs to maintain biological activity under adverse conditions associated with pathogen infection, including drastic pH fluctuations and elevated protease activity [8]. In addition, the characteristic acidic cleft within the TLP structure is believed to contribute to the recognition and interaction with pathogen-derived cell wall components, thereby supporting their antimicrobial activity [9]. Therefore, TLPs are considered important components of the plant defense system and may participate in host resistance during pine wood nematode infection.

Accumulating evidence demonstrates that TLPs can rapidly accumulate to high levels in response to both biotic and abiotic stresses and exhibit pronounced antifungal activity across diverse plant species [10]. For example, over-expression of a *Camellia sinensis CsTLP* gene in potato conferred enhanced resistance to two pathogenic fungi [11]. Transcriptomic analysis of tea plants under gray blight stress further revealed that miRn211-targeted TLP functions as a positive regulator in defense against gray blight disease [12]. In wheat, TaTLP1 was shown to interact with the pathogenesis-related protein TaPR1, thereby enhancing resistance to leaf rust through modulation of reactive oxygen species (ROS) accumulation [13]. Similarly, transgenic rice lines co-expressing rice chitinase *chi11* and tobacco osmotin *ap24* exhibited significantly greater resistance to rice sheath blight than lines expressing either gene alone [14]. Overexpression of a barley *TLP8* gene in rapeseed significantly enhanced the resistance of transgenic lines to the soil-borne pathogen *Plasmodiophora brassicae* [15]. Moreover, heterologous overexpression of the *PpTLP* gene from *Pyrus pyrifolia* in tobacco markedly improved resistance to a broad spectrum of fungal pathogens [16]. A thaumatin-like protein gene *Bx-TLP-1* identified from Pinus massoniana showed dynamic expression changes following *B. xylophilus* infection, and its expression pattern was closely associated with α-pinene accumulation, suggesting that TLPs may participate in host defense responses and pine wood nematode parasitism processes [17].

Although TLP family genes have been extensively characterized in numerous plant species, previous studies in pine and other conifer species have mainly focused on individual defense-related genes or specific stress responses [18,19], and comprehensive genome-wide analyses of the TLP gene family remain limited. In this study, we performed a genome-wide identification of TLP family genes in pine using comprehensive bioinformatics approaches. We analyzed gene number, structural features, conserved motifs, and phylogenetic relationships to elucidate their genomic characteristics. In addition, transcriptomic datasets from multiple time points following pine infection were integrated to examine dynamic expression patterns. Collectively, this study provides the first systematic characterization of the TLP gene family in pine and explores its potential role in resistance to pine wood nematode.

## 2. Materials and Methods

### 2.1. Pine TLP Family Gene Identification

The whole-genome sequence of *Pinus taeda* v2.01 was retrieved from the TreeGenes database (https://treegenesdb.org/). The hidden Markov model (HMM) profile corresponding to the conserved thaumatin domain (PF00314) was downloaded from the Pfam database and used as a query to search the *Pinus taeda* protein dataset using HMMER 3.0 with an E-value threshold of 1 × 10^−5^. Candidate TLPs identified by the HMM search were further verified for the presence of the conserved thaumatin domain using the Pfam and SMART (http://smart.embl-heidelberg.de/) databases, and redundant sequences were manually removed prior to downstream analyses [20]. The presence of the conserved thaumatin domain in each candidate sequence was further validated using the SMART (http://smart.embl-heidelberg.de/) and Pfam databases. Physicochemical properties of the predicted TLPs, including molecular weight and theoretical isoelectric point, were calculated using the ExPASy ProtParam tool (https://web.expasy.org/protparam/, accessed on 15 March 2026). Subcellular localization was predicted using the Plant-mPLoc online server (http://www.csbio.sjtu.edu.cn/bioinf/plant-multi/, accessed on 15 March 2026).

### 2.2. Phylogenetic Analysis of the Pine TLP Gene Family

TLP protein sequences were downloaded from the TreeGenes database (https://cartograplant.org/). Multiple sequence alignment (MSA) was performed using ClustalW with default parameters, including a gap opening penalty of 10, a gap extension penalty of 0.2, and the BLOSUM protein weight matrix for amino acid sequence alignment [21]. Based on the multiple sequence alignment results, a phylogenetic tree was constructed using the maximum likelihood (ML) method implemented in MEGA7.0 with the Jones–Taylor–Thornton (JTT) substitution model and 1000 bootstrap replicates to assess branch support. Other parameters were set to their default values. The resulting phylogenetic tree was subsequently visualized and annotated using the online tool iTOL (https://itol.embl.de/).

### 2.3. Gene Structure, Conserved Motif, and Promoter Cis-Element Analysis

Gene annotation files in GTF format for pine TLP family members were obtained from the TreeGenes database (http://treegenesdb.org/). Exon–intron structures were visualized using TBtools-II [22]. Conserved motifs in TLPs were identified using the MEME (http://meme-suite.org/tools/meme, accessed on 16 March 2026) suite with default parameters. To analyze *cis*-regulatory elements, upstream sequences (promoter regions) of each TLP gene were extracted using TBtools-II. These sequences were submitted to the PlantCARE database to predict putative *cis*-acting regulatory elements. Key regulatory elements were subsequently visualized using TBtools-II [22].

### 2.4. Plant Materials, Nematode Inoculation, and Transcriptome Analysis

Healthy three-year-old *Pinus massoniana* seedlings were selected for nematode inoculation. A hole was drilled downward at a 45 °C angle approximately 10–15 cm above the ground on the main stem. Subsequently, 40 μL of a pine wood nematode suspension (250 nematodes/μL) was injected into the hole using a micropipette. The inoculation site was immediately sealed with parafilm to prevent desiccation. Stem tissues within a 3 cm radius of the inoculation site were collected at 0 h, 6 h, 24 h, and 120 h post-inoculation. Three independent biological replicates were included at each time point. Approximately 1 g of stem tissue per replicate was harvested, immediately frozen in liquid nitrogen, and stored at −80 °C until further analysis. Total RNA samples were shipped on dry ice for eukaryotic transcriptome sequencing. Differential expression analysis was performed using DESeq package (version 1.12.1) from Bioconductor with the |log2(fold change)| > 1 and *p* adjusted < 0.05. To validate the reliability of the RNA-seq results, selected differentially expressed TLP genes were subjected to quantitative real-time PCR (qRT-PCR). Primer pairs were designed using Primer5 software to amplify specific target fragments. Relative gene expression levels were normalized against the internal reference gene *Actin*, which exhibited stable Ct values across all tested samples. qRT-PCR experiments were performed with three biological replicates and relative gene expression levels were calculated using the 2^−ΔΔCt^ method. All primers used in this study are listed in Supplementary Table S4.

### 2.5. Construction of Multilayered Hierarchical Gene Regulatory Networks

A multilayered hierarchical regulatory network associated with pine responses to pine wood nematode infection was constructed using a bottom-up Gaussian graphical model (GGM) algorithm based on transcriptome expression data [23]. Differentially expressed *TLP* genes were designated as bottom-layer target genes, while differentially expressed TFs were screened as potential upstream regulatory candidates. Spearman rank correlation analysis was performed to calculate pairwise gene correlations, while partial correlation analysis was used to evaluate the influence of candidate transcription factors on paired target genes. Regulatory relationships with significant interference effects (*p* < 0.05) were retained for downstream network construction. Multiple testing correction was applied to reduce false-positive associations. The regulatory network was then constructed recursively in a layer-by-layer manner based on the interference strength and connectivity of candidate transcription factors. Finally, the regulatory network was visualized using cytoscape 3.7.1 software.

### 2.6. Yeast One-Hybrid

A yeast one-hybrid (Y1H) assay was performed to verify the binding of a candidate transcription factor to the promoter region of its putative downstream target gene. A MYB recognition sequence containing three tandem repeats was cloned into the pAbAi vector to generate the bait construct. The coding sequence (CDS) of the transcription factor was inserted into the pGADT7 vector to generate the prey construct. Following the lithium acetate transformation protocol described by Gietz and Schiestl [24], the bait construct was first integrated into the Y1H yeast strain. The prey plasmid was subsequently transformed into yeast cells harboring the bait construct. Transformants were plated on SD/-Leu and SD/-Leu/AbA selective media. Yeast growth on selective media was monitored to determine the presence of protein-DNA interactions.

## 3. Results

### 3.1. Identification and Phylogenetic Analysis of TLP Family Genes

**Figure 1:**
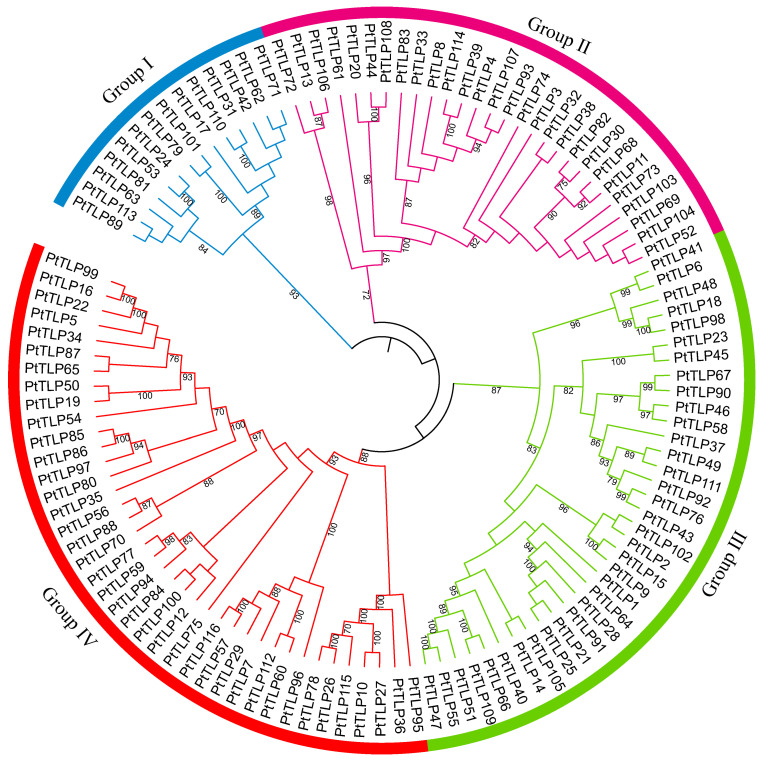
Phylogenetic analysis of TLPs. The phylogenetic tree was constructed using the maximum likelihood method in MEGA7. The phylogenetic tree was constructed using the maximum likelihood (ML) method implemented in MEGA7 based on multiple sequence alignment of full-length TLP protein sequences. Bootstrap analysis was performed with 1000 replicates, and bootstrap support values are indicated at the corresponding nodes. The TLPs were classified into four groups, with each group represented by a different color.

Using a Hidden Markov Model (HMM) search combined with domain verification in the SMART and Pfam databases, a total of 116 putative TLP family genes were identified in the *Pinus taeda* genome. The physicochemical properties of the corresponding TLPs were systematically analyzed (Supplementary Table S1). The predicted molecular weights ranged from 11,745.37 Da (PtTLP85) to 96,557.47 Da (PtTLP76), indicating substantial variation in protein size within the family. The theoretical isoelectric points (pI) varied from 4.03 to 9.38. Notably, 73 of the 116 TLPs exhibited pI values below 7.0, suggesting that acidic members predominate in this family. The aliphatic index ranged from 52.52 to 95.65, implying considerable differences in potential thermostability among members. Analysis of the grand average of hydropathicity (GRAVY) values revealed that 56 proteins displayed positive values, indicating hydrophobic characteristics, whereas 60 proteins had negative values, suggesting an overall hydrophilic nature. Subcellular localization prediction indicated that 102 TLPs were localized to the extracellular, 12 to the plasma membrane, and 1 to the nucleus or chloroplast, suggesting functional diversification across cellular compartments. To elucidate the evolutionary relationships among the pine TLPs, a maximum likelihood (ML) phylogenetic tree was constructed based on the full-length amino acid sequences of the 116 identified members. Phylogenetic analysis clustered these proteins into four distinct groups (Group I–IV) (Figure 1). Among them, Group IV was the largest clade, comprising 39 members, followed by Group III (35 members) and Group II (28 members), whereas Group I represented the smallest clade with 14 members.

### 3.2. Conserved Motif Composition and Gene Structure Characteristics of the TLP Family

**Figure 2:**
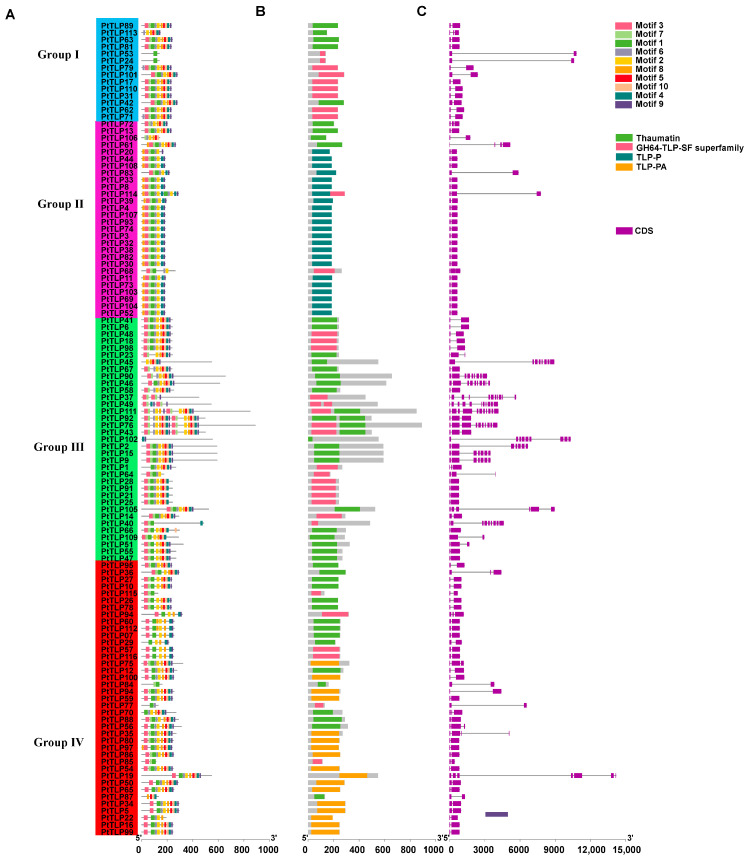
Analysis of gene structures, conserved protein domains, and motifs of pine TLP genes. (**A**), Conserved motif composition of pine TLPs identified using the MEME program. Different colored boxes represent distinct conserved motifs. (**B**), Conserved protein domains of TLPs, different domain types are represented by different colors. (**C**), Gene structure analysis showing exon–intron organization. Purple boxes represent exons and black lines indicate introns.

Conserved motif analysis of TLPs was conducted using the MEME suite, resulting in the identification of 10 conserved motifs (Figure 2A). Most TLP members possess complete or nearly complete motif compositions; however, several members exhibit motif loss. For instance, most genes in Group II lack Motifs 5 and 10, whereas a few proteins, such as PtTLP53 and PtTLP24, retain only Motif 1. Domain analysis revealed that most TLPs contain the canonical thaumatin domain or the GH64-TLP-SF superfamily domain, indicating a high degree of conservation in disease resistance-related functions (Figure 2B). Notably, the TLP-P domain was exclusively detected in Group II members, whereas the TLP-PA domain was specific to Group IV members. Gene structure analysis further demonstrated variation in exon–intron organization among different subgroups (Figure 2C). Group II members generally possess fewer introns and simpler gene structures, while Group III members contain a greater number of introns and exhibit more complex architectures.

### 3.3. Cis-Regulatory Element Analysis of TLP Gene Promoters

**Figure 3:**
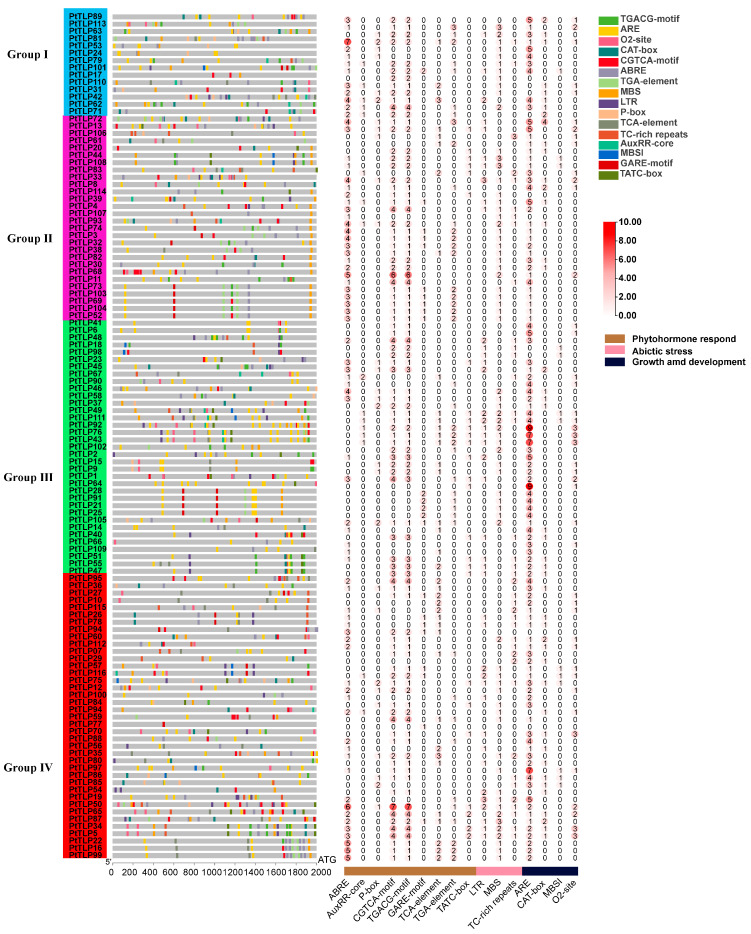
*Cis*-regulatory element analysis of pine TLP gene promoters. The colored ellipses on the right denote different types of *cis*-elements involved in various biological processes. Different colored ellipses on the right represent distinct categories of cis-elements associated with hormone responsiveness, stress responses, light responsiveness, and growth and development. The distribution and frequency of major cis-elements, including ABRE, SA-responsive, and MeJA-responsive elements, and the numbers shown in the figure indicate the frequency of each cis-element identified in the promoter regions of pine TLP genes.

*Cis*-regulatory element analysis revealed that the 2000 bp upstream promoter regions of pine TLP family genes are enriched in diverse regulatory elements associated with light responsiveness, phytohormone signaling, and growth and developmental processes (Figure 3). Light-responsive elements were widely distributed across nearly all TLP members. In addition, abscisic acid (ABA)-, methyl jasmonate (MeJA)-, and gibberellin-responsive elements were frequently detected, suggesting that pine TLP genes may participate in complex hormone-mediated signaling networks. Notably, regulatory elements associated with meristem expression, seed-specific regulation, and cell cycle control were present in several promoters, implying potential roles in developmental regulation.

### 3.4. Differential Expression Analysis of TLP Genes in Response to Pine Wood Nematode Infection

**Figure 4:**
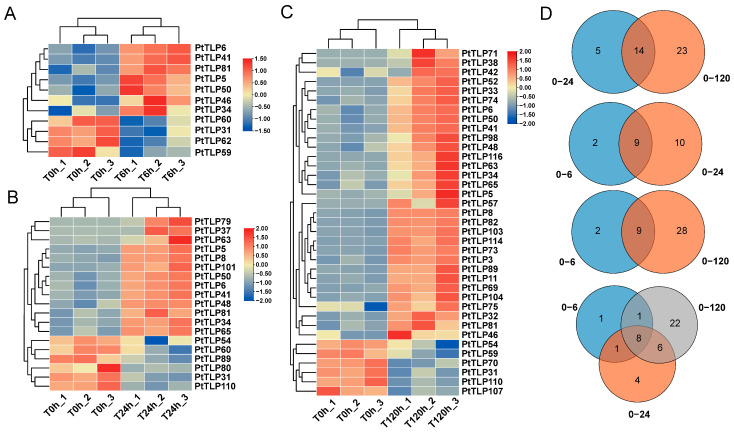
Expression patterns of *TLP* genes under pine wood nematode infection (**A**–**C**), Heatmaps showing DEGs in 6 h, 24 h and 120 h under control and pine wood nematode disease. Color gradients represent relative expression levels based on normalized transcript abundance values, with red indicating upregulated expression and blue indicating downregulated expression. (**D**), Venn diagram illustrating the overlap of DEGs between the different time points. The numbers in each region represent the number of shared or uniquely expressed DEGs.

Based on transcriptome data obtained at four time points (0 h, 6 h, 24 h, and 120 h) following *Pinus massoniana* infection with pine wood nematode, the dynamic expression patterns of TLP family genes were systematically analyzed (Supplementary Table S2). Compared with the control group (0 h), 11 TLP genes were differentially expressed at 6 h post-inoculation, including 7 up-regulated and 4 down-regulated genes (Figure 4A). At 24 h, 19 TLP genes exhibited significant differential expression, of which 13 were up-regulated and 6 were down-regulated (Figure 4B). At 120 h, 37 TLP genes were differentially expressed, with 31 up-regulated and 6 down-regulated (Figure 4C). Comparative analysis of differentially expressed genes (DEGs) across time points revealed substantial overlap. Specifically, nine DEGs were shared between the 0–6 h and 0–24 h comparisons, nine were common to the 0–6 h and 0–120 h comparisons; and fourteen were shared between the 0–24 h and 0–120 h datasets. Notably, eight TLP genes were consistently differentially expressed at all three post-inoculation time points.

### 3.5. qRT-PCR Validation of Differentially Expressed TLP Genes

**Figure 5:**
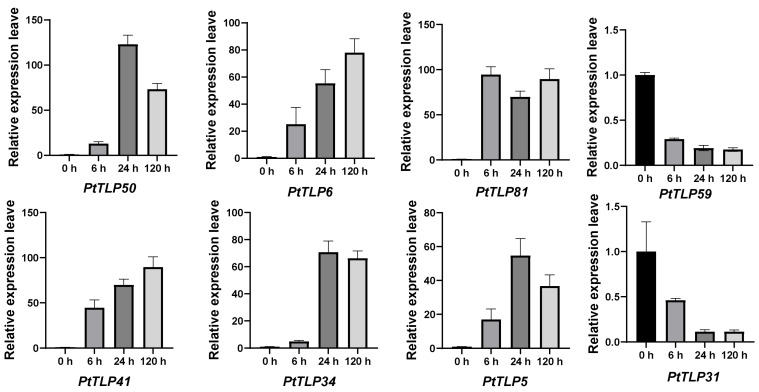
Expression levels of *TLP* genes at different time points following pine wood nematode infection. Gene expression was normalized to the reference gene Actin and calculated using the 2^−ΔΔCt^ method. Data represent the mean ± SD of three independent biological replicates.

To verify the reliability of the RNA-seq results, eight TLP genes that were consistently differentially expressed across the three post-inoculation time points were selected for quantitative real-time PCR (qRT-PCR) analysis. The qRT-PCR results demonstrated that the expression patterns of these genes were largely consistent with the transcriptomic data (Figure 5). This concordance between qRT-PCR and RNA-seq results confirms the accuracy and reliability of the transcriptome analysis.

### 3.6. Construction of a Multilevel Regulatory Network in Response to Pine Wood Nematode Infection

**Figure 6:**
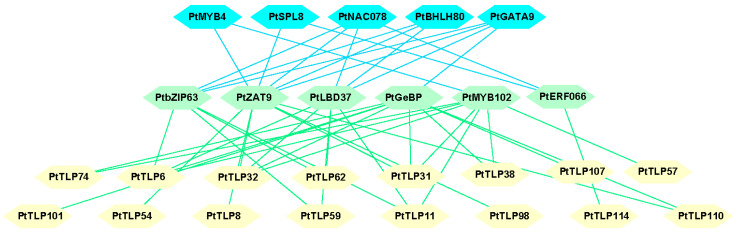
Multilayer transcriptional regulatory network of TLP genes in response to pine wood nematode infection. Blue nodes (top layer) represent first-layer TFs, including members of the MYB, SBP, GATA, NAC, and bHLH families (from left to right). Green nodes (middle layer) represent second-layer TFs, including ERF, MYB, GeBP, C2H2, bZIP, and LBD families (from left to right). Yellow nodes (bottom layer) represent downstream TLP target genes. Edges indicate predicted regulatory relationships between TFs and their target genes.

To elucidate the regulatory mechanisms underlying pine responses to pine wood nematode infection, a multilayered gene regulatory network was constructed using a bottom-up Gaussian graphical model (GGM) algorithm [23]. Differentially expressed *TLP* genes were designated as bottom-layer target genes, while differentially expressed transcription factors (TFs) were screened as potential upstream regulatory candidates (Supplementary Table S3). The resulting network comprised three hierarchical regulatory layers (Figure 6). The first (top) layer included five transcription factors belonging to the MYB, SBP, GATA, NAC, and bHLH families. The second (intermediate) layer consisted of six transcription factors from the ERF, MYB, GeBP, C2H2, bZIP, and LBD families. The third (bottom) layer contained 16 *TLP* genes that may function as downstream effectors in the defense response.

### 3.7. MYB Transcription Factor and MYB-Core Element Interaction Analysis

**Figure 7:**
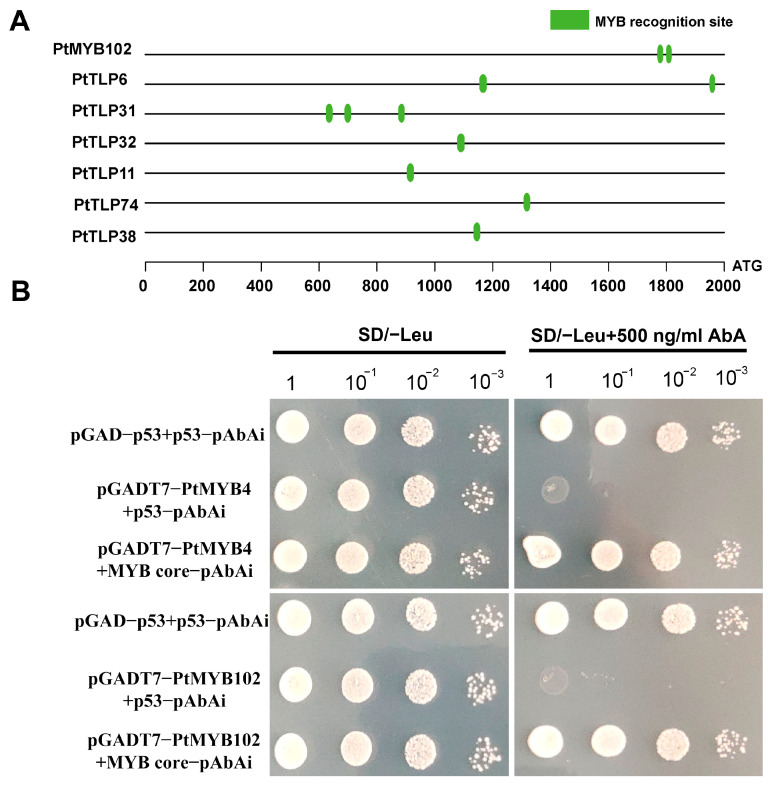
*Cis*-element analysis and yeast one-hybrid validation of MYB transcription factor binding (**A**), Distribution of MYB recognition sites (MYB-core elements) in the promoter regions of the second-layer transcription factor gene *PtMYB102* and six downstream *TLP* genes. Green boxes indicate predicted MYB-core binding motifs along the promoter sequences. (**B**), Y1H assay verifying the binding of PtMYB4 and PtMYB102 to the MYB-core element.

In the multilayer regulatory network constructed in this study, several transcription factor families, including MYB, NAC, bHLH, and GATA, were identified as potential upstream regulators of TLP genes. Studies have shown that the *PtMYB4* gene in the first network may be involved in plant disease resistance, as its homolog in *Arabidopsis thaliana* has been reported to participate in defense responses [25]. Therefore, the MYB transcription factor *PtMYB4* in the first layer was used to verify network regulation. Network predictions indicated that PtMYB4 regulates the second-layer MYB transcription factor *PtMYB102*, which subsequently controls the expression of six *TLP* genes in the third layer (Figure 7A). Promoter sequence analysis revealed that the upstream regions of *PtMYB102* and the six downstream *TLP* genes contain the conserved MYB recognition element (MYB-core), suggesting potential direct transcriptional regulation. Y1H assay results demonstrated that all transformants grew normally on SD/-Leu control medium. However, on SD/-Leu selective medium supplemented with 500 ng/mL aureobasidin A (AbA), only yeast strains co-transformed with the PtMYB4-MYB-core and PtMYB102-MYB-core constructs exhibited normal growth, whereas other combinations failed to grow (Figure 7B). These findings indicate that both PtMYB4 or PtMYB102 can specifically bind to MYB-core *cis*-elements, thereby experimentally validating their hierarchical regulatory relationship within the proposed multilayered transcriptional network.

## 4. Discussion

TLPs, a key subfamily of pathogenesis-related (PR) proteins in plant defense systems, have been identified in a wide range of plant species and are recognized as important components of plant immune responses [6,26]. However, the number of TLP genes varies substantially among species, reflecting lineage-specific expansion and functional diversification during evolution. For example, 81 TLP genes have been identified in *Phyllostachys edulis* [27], 131 in wheat [28], 23 in citrus [29], and 49 in poplar [30]. In the present study, 116 TLP genes were identified in *Pinus taeda*, indicating that the TLP family has undergone considerable expansion in conifers. This expansion may represent an adaptive evolutionary strategy developed during long-term evolution, enabling conifers to better cope with complex environmental conditions and enhancing their resistance to biotic stresses.

Phylogenetic analysis classified pine TLPs into four major clades, with pronounced sequence and structural divergence among them. Members within the same clade generally shared highly similar motif compositions, conserved domain architectures, and exon–intron organization patterns [31]. This structural consistency strongly suggests that genes within each subgroup originated from a common ancestral gene and subsequently expanded through gene duplication events during evolution [32,33]. Most TLPs possessed complete or nearly complete motif compositions, whereas several clades exhibited specific motif losses or domain variations, implying potential functional specialization [34]. Interestingly, among the eight core TLP genes identified during pine wood nematode infection, most were clustered within Group III and Group IV, suggesting that members of these clades may play particularly important roles in pine defense responses. Subcellular localization prediction indicated that most pine TLPs are extracellular, consistent with previous reports in citrus [29] and *Phyllostachys edulis* [27]. Such conserved extracellular localization supports the functional stability of TLPs during evolution. Given that the apoplast serves as a critical interface for sensing pathogen invasion and abiotic stress signals, this localization is well aligned with their role in plant–environment interactions [35,36]. In addition, extracellular localization may facilitate direct interactions between TLPs and pathogen-derived molecules, thereby contributing to early defense perception and apoplastic immune responses during pine wood nematode infection.

*Cis*-regulatory elements within promoter regions play central roles in controlling spatiotemporal gene expression and response intensity [37]. Promoter analysis revealed the widespread presence of stress-responsive and hormone-related cis-elements in pine TLP genes, suggesting that these genes may participate in complex defense signaling networks. In particular, salicylic acid (SA)-responsive elements and methyl jasmonate (MeJA)-responsive elements were highly enriched in promoter regions. SA signaling is generally associated with resistance against biotrophic pathogens and activation of systemic acquired resistance, whereas JA signaling is closely linked to wound responses and defense against necrotrophic pathogens and herbivores [38,39]. Pine wood nematode infection causes severe tissue damage, resin canal disruption, and extensive oxidative stress, which may simultaneously activate multiple hormone signaling pathways. Therefore, the coexistence of SA- and JA-related cis-elements suggests that TLP genes may function as important regulatory nodes integrating multiple defense-related hormone pathways during pine wilt disease progression.

Transcriptome analysis revealed dynamic temporal changes in TLP gene expression during pine wood nematode infection. Several TLP genes were rapidly induced at early infection stages and maintained elevated expression during later stages, indicating that they may participate throughout disease progression. Intersection analysis across time points identified eight consistently differentially expressed genes, which may represent core components of the defense response. Notably, several continuously induced genes belonged to structurally distinct clades, further supporting the possibility of functional diversification among TLP family members during nematode infection. Homology analysis indicated that most of their *Arabidopsis thaliana* orthologs are associated with biotic stress responses. For instance, the *Arabidopsis thaliana* homolog *At1g75040* of *PtTLP5* and *PtTLP34* participates in resistance to Pseudomonas syringae through regulation of SA-mediated signaling [40] and has also been implicated in ATS3-regulated ethylene (ET) and jasmonic acid (JA) pathways, enhancing resistance to *Spodoptera litura* [41]. The homolog *AT4G11650* of *PtTLP6* has been reported to participate in viral infection processes by modulating callose deposition and ROS-mediated defense responses [42]. These findings suggest that pine TLP genes may function in multiple defense-related pathways during pine wood nematode infection. qRT-PCR results further confirmed the reliability of the transcriptomic data, as all eight genes exhibited significant expression changes following infection. However, whether these genes function as resistance factors or are exploited by pathogens requires further functional investigation through overexpression, gene-silencing, or mutant analyses.

Transcriptional regulation is central to plant development. For example, MaERF110 directly binds to the *MaMYB308* promoter, activating its transcription and enhancing susceptibility to Fusarium wilt by reducing lignin deposition [43]. In poplar, PopMYB4 interacts with the histone methyltransferase PopSDG36 to regulate *PopGSTU7* expression via transcriptional repression and H3K36me3 modification, thereby fine-tuning immune responses [44]. PagERF110 inhibits leaf development by direct regulating *PagHB16* in poplar [45]. In this study, a multilayered regulatory network was constructed using a bottom-up Gaussian graphical model (GGM) algorithm, and several transcription factor families, including MYB, SBP, GATA, NAC, and bHLH, were identified as potential upstream regulators of TLP genes. Among them, MYB transcription factors are particularly noteworthy, as they were identified in both the first and second regulatory layers of the network, suggesting that they may function as central regulators coordinating multilayer defense responses. In addition, studies have shown that the Arabidopsis homolog of *PtMYB4* (*AT4G38620.1*) has been reported to regulate metabolic resource allocation during pathogen responses by repressing flavonoid biosynthesis while promoting defense-related metabolite accumulation [25]. The second-layer gene *PtMYB102* corresponds to *Arabidopsis AT4G21440.1*, which is induced by aphid infection and enhances susceptibility through ethylene-dependent signaling by promoting ethylene biosynthesis and suppressing callose deposition [46]. In addition, *AT4G21440.1* is also locally induced upon *Pieris rapae* feeding and contributes to basal resistance by regulating defense-related and cell wall modification genes [47]. These studies demonstrate that homologs of *PtMYB4* and *PtMYB102* are involved in plant biotic stress regulation, supporting their potential key roles in the pine wood nematode response network. Furthermore, cis-element analysis revealed that the promoters of *PtMYB102* and its downstream TLP genes contain MYB-core binding elements, and yeast one-hybrid assays further confirmed the binding ability of MYB transcription factors to MYB-core cis-elements. Nevertheless, comprehensive molecular and genetic experiments such as electrophoretic mobility shift assays (EMSA), dual-luciferase reporter assays or ChIP-qPCR will be required to systematically validate the regulatory relationships proposed in this study.

## 5. Conclusions

In this study, we performed the first systematic genome-wide identification and characterization of the TLP gene family in *P. taeda*. A total of 116 TLP genes were identified and classified into four major clades. Members within the same clade showed highly conserved motifs, domain structures, and exon–intron organization. Promoter analysis revealed abundant stress- and hormone-responsive cis-elements, particularly those associated with SA, MeJA, and MYB-mediated regulatory pathways. Transcriptome profiling and qRT-PCR analyses identified eight core TLP genes that exhibited continuous differential expression throughout the infection process, suggesting that they may play important roles in pine defense responses against *B. xylophilus*. Furthermore, a multilayered regulatory network constructed using the GGM algorithm identified MYB transcription factors as important upstream regulators. Yeast one-hybrid assays further confirmed that PtMYB4 and PtMYB102 specifically bind to MYB-core cis-elements, supporting their potential hierarchical regulatory relationships in TLP-mediated defense pathways. These findings provide new insights into the molecular mechanisms underlying pine responses to pine wood nematode infection and offer valuable candidate genes for future molecular breeding and genetic engineering aimed at improving pine wilt disease resistance. However, this study mainly relied on bioinformatics prediction, transcriptome analysis, and preliminary experimental validation. Additional functional studies, including overexpression, gene-silencing, EMSA, dual-luciferase reporter assays, and gene-editing analyses, will be necessary to further validate the biological functions and regulatory mechanisms of these TLP genes.

## Data Availability

The raw sequence data reported in this paper have been deposited in the Genome Sequence Archive (Genomics, Proteomics & Bioinformatics 2025) in National Genomics Data Center (Nucleic Acids Res 2025), China National Center for Bioinformation/Beijing Institute of Genomics, Chinese Academy of Sciences (GSA: CRA039775) that are publicly accessible at https://ngdc.cncb.ac.cn/gsa (accessed on 11 March 2026). We have made the necessary revisions in the manuscript.

## Supplementary Materials

The following supporting information can be downloaded at: https://www.mdpi.com/article/10.3390/biology15110878/s1, Table S1: The list of 103 TLP genes identified in this article; Table S2: Expression levels of TLP genes under pine wood nematode infection; Table S3: Expression data of transcription factors and TLP genes used for GGM algorithm analysis; Table S4: The primers sequences used in this study; Table S5: The original Ct values of the qRT-PCR experiment.

## References

[1] Li M., Li H., Ding X., Wang L., Wang X., Chen F. (2022). The detection of pine wilt disease: A literature review. Int. J. Mol. Sci..

[2] Back M.A., Bonifácio L., Inácio M.L., Mota M., Boa E. (2024). Pine wilt disease: A global threat to forestry. Plant Pathol..

[3] Nickle W.R., Golden A.M., Mamiya Y., Wergin W.P. (1981). On the Taxonomy and Morphology of the Pine Wood Nematode, Bursaphelenchus xylophilus (Steiner & Buhrer 1934) Nickle 1970. J. Nematol..

[4] Kojima K., Kamuyo A., Masumori M., Sasaki S. (1994). Cellulase activities of pine-wood nematode isolates with different virulences. J. Jpn. For. Soc..

[5] Zribi I., Ghorbel M., Brini F. (2021). Pathogenesis Related Proteins (PRs): From Cellular Mechanisms to Plant Defense. Curr. Protein Pept. Sci..

[6] de Jesús-Pires C., Ferreira-Neto J.R., Bezerra-Neto J.P., Kido E.A., de Oliveira Silva R.L., Pandolfi V., Wanderley-Nogueira A.C., Binneck E., da Costa A.F., Pio-Ribeiro G. (2020). Plant thaumatin-like proteins: Function, evolution and biotechnological applications. Curr. Protein Pept. Sci..

[7] Bano N., Aalam S., Bag S.K. (2022). Tubby-like proteins (TLPs) transcription factor in different regulatory mechanism in plants: A review. Plant Mol. Biol..

[8] Misra R.C., Sandeep, Kamthan M., Kumar S., Ghosh S. (2016). A thaumatin-like protein of Ocimum basilicum confers tolerance to fungal pathogen and abiotic stress in transgenic Arabidopsis. Sci. Rep..

[9] Zhang J., Wang F., Liang F., Zhang Y., Ma L., Wang H., Liu D. (2018). Functional analysis of a pathogenesis-related thaumatin-like protein gene TaLr35PR5 from wheat induced by leaf rust fungus. BMC Plant Biol..

[10] Petre B., Major I., Rouhier N., Duplessis S. (2011). Genome-wide analysis of eukaryote thaumatin-like proteins (TLPs) with an emphasis on poplar. BMC Plant Biol..

[11] Acharya K., Pal A.K., Gulati A., Kumar S., Singh A.K., Ahuja P.S. (2013). Overexpression of Camellia sinensis thaumatin-like protein, CsTLP in potato confers enhanced resistance to Macrophomina phaseolina and Phytophthora infestans infection. Mol. Biotechnol..

[12] Wang S., Liu L., Mi X., Zhao S., An Y., Xia X., Guo R., Wei C. (2021). Multi-omics analysis to visualize the dynamic roles of defense genes in the response of tea plants to gray blight. Plant J..

[13] Wang F., Yuan S., Wu W., Yang Y., Cui Z., Wang H., Liu D. (2020). TaTLP1 interacts with TaPR1 to contribute to wheat defense responses to leaf rust fungus. PLoS Genet..

[14] Sripriya R., Parameswari C., Veluthambi K. (2017). Enhancement of sheath blight tolerance in transgenic rice by combined expression of tobacco osmotin (ap 24) and rice chitinase (chi 11) genes. Vitr. Cell. Dev. Biol.-Plant.

[15] Reiss E., Schubert J., Scholze P., Krämer R., Sonntag K. (2009). The barley thaumatin-like protein Hv-TLP8 enhances resistance of oilseed rape plants to Plasmodiophora brassicae. Plant Breed..

[16] Liu D., He X., Li W., Chen C., Ge F. (2012). Molecular cloning of a thaumatin-like protein gene from Pyrus pyrifolia and overexpression of this gene in tobacco increased resistance to pathogenic fungi. Plant Cell Tissue Organ Cult. (PCTOC).

[17] Meng F., Li Y., Liu Z., Feng Y., Wang X., Zhang X. (2022). Expression of the Thaumatin-Like Protein-1 Gene (Bx-tlp-1) from Pine Wood Nematode Bursaphelenchus xylophilus Affects Terpene Metabolism in Pine Trees. Phytopathology.

[18] Meng F.-L., Wang J., Wang X., Li Y.-X., Zhang X.-Y. (2017). Expression analysis of thaumatin-like proteins from Bursaphelenchus xylophilus and Pinus massoniana. Physiol. Mol. Plant Pathol..

[19] Liu J.-J., Zamani A., Ekramoddoullah A.K. (2010). Expression profiling of a complex thaumatin-like protein family in western white pine. Planta.

[20] Finn R.D., Clements J., Eddy S.R. (2011). HMMER web server: Interactive sequence similarity searching. Nucleic Acids Res..

[21] Thompson J.D., Higgins D.G., Gibson T.J. (1994). CLUSTAL W: Improving the sensitivity of progressive multiple sequence alignment through sequence weighting, position-specific gap penalties and weight matrix choice. Nucleic Acids Res..

[22] Chen C., Wu Y., Li J., Wang X., Zeng Z., Xu J., Liu Y., Feng J., Chen H., He Y. (2023). TBtools-II: A “one for all, all for one” bioinformatics platform for biological big-data mining. Mol. Plant.

[23] Kumari S., Deng W.P., Gunasekara C., Chiang V., Chen H.S., Ma H., Davis X., Wei H.R. (2016). Bottom-up GGM algorithm for constructing multilayered hierarchical gene regulatory networks that govern biological pathways or processes. Bmc Bioinform..

[24] Gietz R.D., Schiestl R.H. (1991). Applications of high efficiency lithium acetate transformation of intact yeast cells using single-stranded nucleic acids as carrier. Yeast.

[25] Schenke D., Böttcher C., Scheel D. (2011). Crosstalk between abiotic ultraviolet-B stress and biotic (flg22) stress signalling in Arabidopsis prevents flavonol accumulation in favor of pathogen defence compound production. Plant Cell Environ..

[26] Cao J., Lv Y., Hou Z., Li X., Ding L. (2016). Expansion and evolution of thaumatin-like protein (TLP) gene family in six plants. Plant Growth Regul..

[27] Gu Y., Yu H., He S., Zhang P., Ma X. (2023). Genome-Wide Identification and Characterization of the TLP Gene Family in Phyllostachys edulis and Association with Witches’ Broom Disease Resistance in Bamboo. Int. J. Mol. Sci..

[28] Gao Z., Sun M., Shao C., Chen Y., Xiang L., Wu J., Wang J., Chen X. (2024). Genome-wide analysis and characterization of the TaTLP gene family in wheat and functional characterization of the TaTLP44 in response to Rhizoctonia cerealis. Plant Physiol. Biochem..

[29] Li X., Fan L., Liu C., Wang X., Zhang X., Tong X. (2025). Genome-Wide Identification and Biotic Stress Responses of TLP Gene Family in Citrus sinensis. Int. J. Mol. Sci..

[30] Guo M., Ma X., Xu S., Cheng J., Xu W., Elsheery N.I., Cheng Y. (2024). Genome-Wide Identification of TLP Gene Family in Populus trichocarpa and Functional Characterization of PtTLP6, Preferentially Expressed in Phloem. Int. J. Mol. Sci..

[31] Liu D., Jiang L., Jiang L., Zhou T., Wu Y., Ma J., Liu Y., Zhang X. (2025). ZoERF60 enhances antioxidant defense and osmotic homeostasis for heat and humidity resilience in ginger. Plant Sci..

[32] Blanc G., Wolfe K.H. (2004). Widespread paleopolyploidy in model plant species inferred from age distributions of duplicate genes. Plant Cell.

[33] Flagel L.E., Wendel J.F. (2009). Gene duplication and evolutionary novelty in plants. New Phytol..

[34] Bailey T.L., Boden M., Buske F.A., Frith M., Grant C.E., Clementi L., Ren J., Li W.W., Noble W.S. (2009). MEME SUITE: Tools for motif discovery and searching. Nucleic Acids Res..

[35] Qi Y., Tsuda K., Glazebrook J., Katagiri F. (2011). Physical association of pattern-triggered immunity (PTI) and effector-triggered immunity (ETI) immune receptors in Arabidopsis. Mol. Plant Pathol..

[36] O’Brien J.A., Daudi A., Butt V.S., Bolwell G.P. (2012). Reactive oxygen species and their role in plant defence and cell wall metabolism. Planta.

[37] Higo K., Ugawa Y., Iwamoto M., Korenaga T. (1999). Plant cis-acting regulatory DNA elements (PLACE) database: 1999. Nucleic Acids Res..

[38] Vlot A.C., Dempsey D.M.A., Klessig D.F. (2009). Salicylic acid, a multifaceted hormone to combat disease. Annu. Rev. Phytopathol..

[39] Wasternack C., Hause B. (2013). Jasmonates: Biosynthesis, perception, signal transduction and action in plant stress response, growth and development. An update to the 2007 review in Annals of Botany. Ann. Bot..

[40] Xu B., Fan B., Chen Z. (2024). A critical role of a plant-specific TFIIB-related protein, BRP1, in salicylic acid-mediated immune response. Front. Plant Sci..

[41] Savadogo E.H., Shiomi Y., Yasuda J., Akino T., Yamaguchi M., Yoshida H., Umegawachi T., Tanaka R., Suong D.N.A., Miura K. (2021). Gene expression of PLAT and ATS3 proteins increases plant resistance to insects. Planta.

[42] He R., Li Y., Bernards M.A., Wang A. (2025). Turnip mosaic virus selectively subverts a PR-5 thaumatin-like, plasmodesmal protein to promote viral infection. New Phytol..

[43] Li Y., Hu Y., Xiao W., Yan L., Feng J., Cao M., Si Y., Lyu J., Zhao Y., Li K. (2026). A MaERF110-MaMYB308 Transcriptional Module Negatively Regulates Lignin-Mediated Defence Against Fusarium Wilt in Banana. Plant Biotechnol. J..

[44] Tan S., Guo H., Wang H., Wu J., Liu L., Bao F., Xie J. (2026). PopMYB4 orchestrates disease resistance through H3K36me3-mediated epigenetic activation of PopGSTU7 in poplars. New Phytol..

[45] Cheng Z., Zhu Y., He X., Fan G., Jiang J., Jiang T., Zhang X. (2025). Transcription factor PagERF110 inhibits leaf development by direct regulating PagHB16 in Poplar. Plant Sci..

[46] Zhu L., Guo J., Ma Z., Wang J., Zhou C. (2018). Arabidopsis Transcription Factor MYB102 Increases Plant Susceptibility to Aphids by Substantial Activation of Ethylene Biosynthesis. Biomolecules.

[47] De Vos M., Denekamp M., Dicke M., Vuylsteke M., Van Loon L., Smeekens S.C., Pieterse C.M. (2006). The Arabidopsis thaliana Transcription Factor AtMYB102 Functions in Defense Against the Insect Herbivore Pieris rapae. Plant Signal. Behav..

